# ART Suppresses Plasma HIV-1 RNA to a Stable Set Point Predicted by Pretherapy Viremia

**DOI:** 10.1371/journal.ppat.0030046

**Published:** 2007-04-06

**Authors:** Frank Maldarelli, Sarah Palmer, Martin S King, Ann Wiegand, Michael A Polis, JoAnn Mican, Joseph A Kovacs, Richard T Davey, Diane Rock-Kress, Robin Dewar, Shuying Liu, Julia A Metcalf, Catherine Rehm, Scott C Brun, George J Hanna, Dale J Kempf, John M Coffin, John W Mellors

**Affiliations:** 1 HIV Drug Resistance Program, National Cancer Institute, Frederick, Maryland, United States of America; 2 Global Pharmaceutical Research and Development, Abbott Laboratories, Abbott Park, Illinois, United States of America; 3 Lab Immunoregulation, National Institute of Allergy and Infectious Diseases, National Institutes of Health, Bethesda, Maryland, United States of America; 4 Department of Critical Care, National Institutes of Health Clinical Center, National Institutes of Health, Bethesda, Maryland, United States of America; 5 SAIC-Frederick, Frederick, Maryland, United States of America; 6 Division of Infectious Diseases, University of Pittsburgh Medical Center, Pittsburgh, Pennsylvania, United States of America; King's College London, United Kingdom

## Abstract

Current antiretroviral therapy is effective in suppressing but not eliminating HIV-1 infection. Understanding the source of viral persistence is essential for developing strategies to eradicate HIV-1 infection. We therefore investigated the level of plasma HIV-1 RNA in patients with viremia suppressed to less than 50–75 copies/ml on standard protease inhibitor- or non-nucleoside reverse transcriptase inhibitor-containing antiretroviral therapy using a new, real-time PCR-based assay for HIV-1 RNA with a limit of detection of one copy of HIV-1 RNA. Single copy assay results revealed that >80% of patients on initial antiretroviral therapy for 60 wk had persistent viremia of one copy/ml or more with an overall median of 3.1 copies/ml. The level of viremia correlated with pretherapy plasma HIV-1 RNA but not with the specific treatment regimen. Longitudinal studies revealed no significant decline in the level of viremia between 60 and 110 wk of suppressive antiretroviral therapy. These data suggest that the persistent viremia on current antiretroviral therapy is derived, at least in part, from long-lived cells that are infected prior to initiation of therapy.

## Author Summary

Combination antiretroviral therapy is effective in reducing, but not eliminating, HIV-1 replication. Residual viremia during suppressive antiretroviral therapy may arise from a number of sources, including reservoirs of long-lived virus-producing cells, or ongoing complete cycles of viral replication. Here, we used a new, more sensitive assay of HIV-1 RNA to measure residual viremia in a large cohort of patients with prolonged suppression on antiretroviral therapy. We found a persistent, stable level of viremia in patients on prolonged therapy that correlated with pretherapy levels of HIV-1. Over 80% of patients had viremia ≥1 copy/ml plasma, and the level of viremia was independent of the drug regimen patients were taking. These data strongly suggest that persistent viremia on antiretroviral therapy is likely derived from reservoirs of long-lived virus-producing cells that are not affected by currently available drugs that target new cycles of viral replication. New antiviral strategies that eradicate this reservoir will be necessary to cure HIV-1 infection.

## Introduction

Infection with HIV-1 results in progressive immunodeficiency and death from opportunistic infection or cancer. Current antiretroviral therapy is effective in suppressing plasma viremia to levels below the detection limit of FDA-approved assays (50–75 copies HIV-1 RNA/ml), restoring immune function, and reducing morbidity and mortality. Antiretroviral therapy does not cure HIV-1 infection, however, and, at a minimum, eradication of HIV-1 infection will require complete suppression of its replication as well as elimination of latent viral reservoirs. Although some reports have shown the persistence of low-level viremia in patients on suppressive therapy [1–6], the determinants of this viremia, and whether it results from ongoing replication cycles or release from latent reservoirs, are not well defined [2,7–9] due in part to the limited sensitivity of prior HIV-1 RNA assays. To investigate these issues, we developed a real-time PCR-based method (single-copy assay, SCA) capable of detecting and reliably quantifying HIV-1 RNA with a limit of one copy per ml plasma [10]. Using this assay, we have found that more than 80% of patients on currently recommended antiretroviral therapies have quantifiable viremia for at least 2 y after initiation of therapy. These results have important implications for understanding the mechanism of HIV-1 persistence despite long-term antiretroviral therapy.

## Results/Discussion

**Figure 1:**
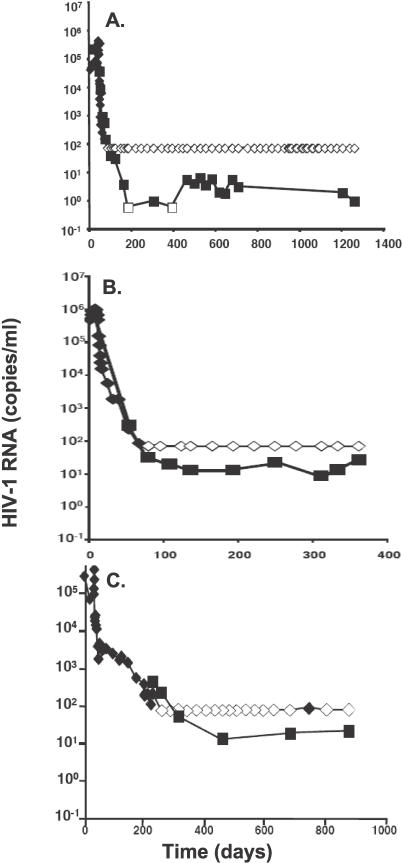
Decline and Stable Persistence of Plasma HIV-1 RNA in Patients Suppressed on Antiretroviral Therapy Plasma from patients initiating antiretroviral therapy with stavudine + lamivudine + efavirenz (patients A and B) or stavudine + lamivudine + indinavir + nevirapine (patient C) was assayed by bDNA (diamonds) and SCA (squares). Levels of virus above the limit of detection for each assay are shown by filled symbols; levels below this limit are shown by hollow symbols plotted at the assay limit.

We first measured plasma HIV-1 RNA with both an FDA-approved assay (bDNA; detection limit 75 copies/ml) and SCA in three patients initiating antiretroviral therapy. As expected [11–13], therapy produced a rapid decline in plasma HIV-1 RNA (Figure 1), reaching undetectable levels within 50–260 d. HIV-1 RNA values from the two assays were similar at levels that were detectable by both assays, but the SCA continued to detect HIV-1 RNA throughout the sampling period, well below the limit of detection of the bDNA assay.

**Figure 2:**
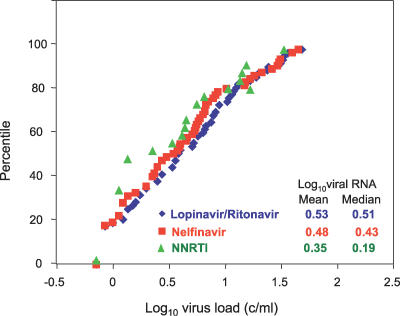
Distribution of Plasma HIV-1 RNA Levels in Patients with Persistently Suppressed Viremia (HIV-1 RNA <50 Copies/ml from Week 24 to 60) on Standard Antiretroviral Therapy Week 60 viral RNA levels for M98–863 patients on LPV/r- (blue) or NFV-containing (red) regimens were determined by SCA. The percentile of patients in each group with a given RNA level is presented. SCA determinations for patients on NNRTI- containing regimens (*n* = 28) for >1 y are included (green). The inset shows the mean and median log_10_ RNA levels for each group.

These initial observations suggested that low-level viremia can persist for years in patients receiving suppressive antiretroviral therapy. To investigate this phenomenon in a larger patient population and to compare the effects of different treatment regimens, we analyzed specimens from study M98–863, a Phase III randomized clinical trial comparing lopinavir/ritonavir (LPV/r) and nelfinavir (NFV), each in combination with stavudine and lamivudine, in previously antiretroviral-naïve HIV-1-infected individuals [14]. We studied a subset of 145 patients (see Figure S1) whose plasma HIV-1 RNA declined to less than 50 copies/ml within 24 wk of initiating therapy and remained at this level at all time points through 60 wk. SCA results were available from 130 patients (63 on LPV/r and 67 on NFV). As shown in Figure 2, HIV-1 RNA values at week 60 in the two arms combined ranged from <0.6–174 copies/ml with a geometric mean (median) of 3.2 (3.1) copies/ml and with no significant between-arm differences in the mean (*p* = 0.56) or overall distribution of values (*p* = 0.82). Plasma HIV-1 RNA was below the limit of quantification by SCA in about 17% of patients (LPV/r arm: 17%; NFV arm: 18%).

We also determined the distribution of HIV-1 RNA levels in 28 patients enrolled in National Institutes of Health (NIH) studies whose viremia was suppressed to <75 copies/ml on non-nucleoside reverse transriptase inhibitor (NNRTI)-containing regimens. As shown in Figure 2, the distribution of SCA values from these patients was comparable to that of patients in the M98–863 trial (*p* = 0.22); specifically, the mean values were not significantly different (*p* = 0.17) and there was no difference between efavirenz- and nevirapine-containing regimens (*p* = 0.29).

**Figure 3:**
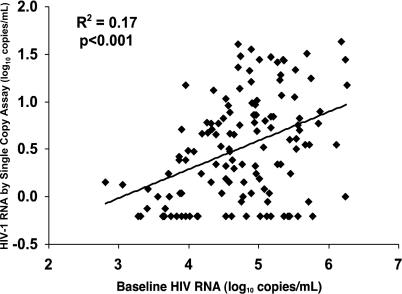
Correlation between Pretherapy and On-Therapy Viral RNA Levels Pretherapy and week 60 SCA values were determined for 130 patients from both the NFV and LPV/r arms in M98–863. Correlation coefficients for patients taking LPV/r and NFV regimens were similar.

We investigated virologic, immunologic, demographic, and clinical parameters for correlates of persistent viremia in samples from both arms of the M98–863 trial. Comparison of the HIV-1 RNA level by SCA at week 60 to pretherapy RNA levels (Figure 3) showed a significant correlation (*p* < 0.001) and a median decrease between pretherapy and week 60 HIV-1 RNA values of 14,250-fold (range 448- to1,720,000-fold). The correlation coefficient (*r* ^2^ = 0.17) suggests pretherapy viral RNA level accounts for 17% of the variability in stable viremia on therapy, and that other factors, such as time since infection, may contribute to the level of on-therapy viremia. No significant differences between treatment arms were detected in median HIV-1 RNA reduction, and additional analyses (unpublished data) indicated that both the magnitude of HIV-1 RNA reduction and the level of persistent viremia were not associated with pretherapy CD4+ T-cell count, change in CD4+ T-cell count on therapy, age at enrollment, or gender.

**Figure 4:**
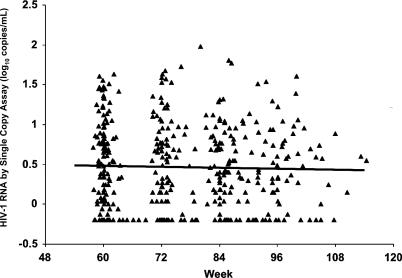
HIV-1 RNA Levels Over 50 wk of Suppressive Antiretroviral Therapy Plasma samples from patients in both arms of the M98–863 trial with viremia suppressed to <50 copies/ml during weeks 60–110 were subjected to SCA analysis. HIV-1 RNA levels for each patient are presented here as a function of weeks on therapy.

To examine the stability of the low level of viremia in the M98–863 study, we next performed a longitudinal analysis of HIV-1 RNA after week 60. Of the 145 patients with pretherapy samples who had suppression of plasma HIV-1 RNA between weeks 24 and 60, 117 had samples available and continuous suppression (all values <50 copies/ml, Figure S1) at subsequent time points. Results of SCA testing of one to five samples (median of three) from each of these patients during weeks 60–110 of therapy are shown in Figure 4. No significant decreases in HIV-1 RNA levels were detected over time, regardless of whether patients were analyzed overall or separately by treatment group; i.e., the HIV-1 RNA slopes were not significantly different from 0 overall or in either treatment group. Among all patients combined, a half-life of less than 67 wk could be excluded with 95% confidence, using a mixed model linear regression analysis.

The study presented here represents the first large-scale and long-term analysis of persistent viremia in patients on standard antiretroviral therapy. Residual viremia on therapy (median viral RNA of 3.1 copies/ml plasma) was more than 10-fold below the limit of detection of FDA-approved HIV-1 RNA assays. Viremia was suppressed to less than 0.6 copies/ml in approximately one-fifth of patients, nearly 100-fold lower than the detection limit of FDA-approved assays. As such, SCA permitted a more accurate estimate of the maximal effect of antiretroviral therapy. Comparison of pre- and post-therapy levels revealed a median reduction in HIV-1 RNA of more than 14,000-fold. After this maximal reduction in viremia occurred, however, HIV-1 RNA levels remained stable with no significant decline between the first and second years of therapy.

In previous studies, investigators using smaller datasets have reported slow declines in plasma HIV-1 RNA corresponding to half-lives of infected cells of 6 mo [1,6] or longer [8,15]. Our longitudinal analysis, using a much larger dataset, revealed no significant evidence of decline of HIV-1 RNA levels between 60 and 110 wk of therapy. It is possible, however, that a slow decline in HIV-1 RNA with half-life of greater than 67 wk could have been missed with the current dataset. We are currently assembling a new set of samples with longer follow-up to investigate this possibility.

Three sources of persistent viremia have been proposed: (i) ongoing cycles of viral replication in the presence of antiretroviral drugs because of inadequate drug inhibitory potency; (ii) production of HIV-1 from sanctuary sites into which antiretrovirals do not penetrate sufficiently; or (iii) reservoirs of long-lived cells infected prior to the initiation of therapy with production of HIV-1 from integrated proviruses, but without complete cycles of replication occurring because of blockade by antiretroviral drugs [8,15–17]. The marked stability of viremia detected in this study, in addition to the similar levels of suppression by LPV/r-, NFV-, or NNRTI-containing regimens, strongly suggests that current antiretroviral therapies inhibit HIV-1 replication cycles and virus production to below a background level set by virus released from long-lived cells. More specifically, the residual viremia is likely derived from a reservoir of long-lived cells infected before the initiation of therapy. This reservoir might comprise chronically infected cells that produce virus at a low stable rate and/or latently-infected cells, like resting CD4+ T cells, that can be isolated from infected individuals on suppressive therapy and activated to produce infectious virus [8,15,18,19]. Our data describe only the level of viremia, not other viral characteristics. Others have reported no accumulation of new resistance mutations and little genetic variation over time in patients with suppressed viremia. Recent analyses have suggested that a limited number of genetic variants circulate during suppression [20–22], also consistent with a model of HIV-1 production from long-lived cells. Analyses of HIV-1 replication during suppressive antiretroviral therapy intended to distinguish between active replication cycles and production from reservoirs have yielded conflicting results [6,23,24], and additional treatment intensification trials to distinguish these possibilities are underway (NIH study 02-I-0232, http://clinicalstudies.info.nih.gov/cgi/protinstitute.cgi?NIAID.0.html). Information from such trials is essential in designing future antiretroviral strategies to fully suppress viremia.

This study analyzed only those participants in the M98–863 study whose viremia remained suppressed to <50 copies/ml for 60–110 wk. As demonstrated in prior intent-to-treat analyses of M98–863, LPV/r had superior efficacy compared to NFV as judged by Kaplan-Meier analysis of plasma HIV-1 RNA rebound to greater than 400 copies/ml through 96 wk [14,25–27]. The present results imply that the difference in efficacy is not associated with differences in residual viremia in the two groups. Early therapeutic failures have been hypothesized to result from the emergence of mutations that exist prior to initiation of antiretroviral therapy [16,28]. In this regard, the superiority of LPV/r may be related in part to its contribution to a higher genetic barrier to resistance for the combination regimen, as compared to NFV, for which a single mutation confers significant resistance [29,30]. Other factors that may have contributed to the differential efficacy observed between LPV/r and NFV are related to differences in pharmacokinetic profiles, as lopinavir plasma concentrations exceed the IC_50_ for wild-type HIV by a considerably greater margin than observed with NFV. Consequently, individual pharmacokinetic variability and adherence lapses may be more likely to result in loss of suppression with NFV-based regimens compared to LPV/r-based therapy. Similar levels of persistent viremia in patients undergoing NNRTI-containing therapy for prolonged periods (mean = 111.2 wk) were noted even though the genetic barrier to NNRTI is likely to be low; again, early drug failures due to pre-existing mutations were likely excluded from selection. The presence of persistent viremia has a number of important implications for development and application of antiretroviral therapies. Few patients on antiretroviral therapy have “undetectable” viremia if sufficiently sensitive assays are employed, and greater suppression of viremia can probably not be achieved using current inhibitors of HIV-1 replication because persistent viremia likely arises, at least in part, from chronically infected cells. New therapeutic strategies will be needed to eliminate persistent viremia and its source.

## Materials and Methods

### Research participants.

Plasma samples were obtained from stored specimens from patients with plasma HIV-1 RNA <50 copies/ml in the M98–863 trial and from patients on treatment at the NIH Clinical Center as follows.

### M98–863 patients.

M98–863 was a randomized double-blind, Phase III study comparing LPV/r (400/100 mg twice daily) (*n* = 326) with NFV (750 mg three times daily or 1,250 mg twice daily) (*n* = 327), each in combination with stavudine and lamivudine, in previously antiretroviral-naïve HIV-1-infected patients [14]. All patients in M98–863 who achieved plasma HIV-1 RNA values <50 copies/ml by week 24 of therapy and who remained undetectable at that level at all study visits through week 60 of follow-up were identified for analysis (*n* = 237). From this group, a subset of 163 participants treated at investigational sites in North America was identified for SCA testing. Of these, six were excluded for lack of archived samples and 12 for inefficient amplification of baseline sample. Pretherapy samples of 145 participants were amplifiable by SCA (Figure S1), and only these participants were candidates for further analyses. Of these, 130 also had valid assay results for week 60 samples and constituted the week 60 group analysis. All 145 participants with amplifiable pretherapy samples who remained on study and maintained suppressed plasma HIV-1 RNA levels <50 copies/ml through 110 wk of follow-up were considered for longitudinal analysis. As shown in Figure S1, six participants did not have samples available after week 60; 21 did not maintain complete suppression after week 60 by conventional viral RNA testing; and one had no valid assay results during analysis. Therefore, 117 participants were included in the longitudinal analysis.

Prior to participation in the study, all M98–863 patients provided informed consent for viral RNA quantitation. The protocols and procedures for subsequent SCA analysis of these samples by the HIV Drug Resistance Program were reviewed and approved by the NIAID IRB.

### Patients taking NNRTI-containing regimens.

Patients taking NNRTI-containing regimens with suppressed plasma HIV-1 RNA levels by FDA-approved testing were also recruited from treatment studies performed at the NIH Clinical Center (*n* = 28). All patients were taking Department of Health and Human Services (DHHS) guideline-approved antiretroviral regimens containing efavirenz (*n* = 22) or nevirapine (*n* = 6) and were either sampled frequently by bDNA or SCA following initiation of therapy or had been on therapy for 20 to >250 wk (mean 111.2 wk) prior to SCA sampling. All patients were well and without history of recent intercurrent illness at the time of phlebotomy, had hemoglobin levels ≥12 g/dl, and provided informed consent for phlebotomy and for research sample storage.

### HIV-1 RNA determination.

The SCA for HIV-1 RNA detection was performed as described previously [10], starting with 7 ml plasma, except for the M98–863 samples, from which only 3 ml plasma was available. As a result, the lower limit of quantitation for these samples was 0.6 copies HIV-1 RNA/ml, compared to 0.3 copies/ml when 7 ml plasma was used. In all analyses, values below the assay quantitation limit for study M98–863 (0.6 copies/mL) were considered to be equal to the assay limit. Plasma was obtained from whole blood samples within 2–4 h of phlebotomy and immediately frozen at −70 °C. [10,31]. For each sample, three separate aliquots of the cDNA product were assayed for HIV-1 RNA and two aliquots for the recombinant avian retrovirus internal standard RNA using real-time PCR of conserved sequences within *gag* as described [10]. About 10% of M98–863 patients were excluded because SCA and Amplicor assays on pretherapy samples were significantly discordant due to inefficient amplification by SCA (Figure S2), probably a result of polymorphism in the probe or primer sequences (A. Wiegand and S. Palmer, unpublished data). In all other samples, there was a close correlation between commercial Amplicor RT-PCR and SCA values for pretherapy samples (*r* ^2^ = 0.61, Figure S2). Further details of extraction, optimum amplification conditions, and performance characteristics, as well as quality control procedures to prevent artifactual amplification, have been described [10].

Study M98–863 used the PCR-based Amplicor HIV-1 MONITOR assay version 1.0 for plasma HIV-1 RNA quantitation, performed according to manufacturer's specifications (Roche Diagnostics, http://www.roche.com). For patients taking NNRTI-containing regimens, plasma HIV-1 RNA was quantified using the bDNA-based VERSANT HIV-1 RNA assay version 3.0 according to manufacturer's specifications (Bayer Diagnostics, http://diagnostics.siemens.com) in a manufacturer-certified site using the semiautomated Bayer System 340 unit. Versant HIV-1 RNA version 3.0 is approved by the FDA for clinical use with a limit of quantification of 75 copies HIV-1 RNA/ml plasma, although our reported experience with this assay indicates a quantification limit of 50 copies/ml [32,33].

### Statistical analysis.

For log-transformed baseline viral RNA determinations, comparisons between Amplicor HIV-1 RNA assays and SCA were conducted using linear regression and Pearson's correlation, as were comparisons between log-transformed viral RNA determinations at baseline and week 60. Mean SCA values were compared between groups using a one-way analysis of variance; comparisons of distributions of SCA values were conducted using the Kolmogorov-Smirnov test. A linear mixed effects regression model with a spatial linear correlation structure to account for correlation between repeated measurements within participants was used to assess the relationship between time and log-transformed viral determinations using SCA. In all analyses, values below the assay quantitation limit for study M98–863 (0.6 copies/mL) were considered to be equal to the assay limit. Sensitivity analyses using other imputation methods did not alter results meaningfully.

## Supporting Information

Figure S1 Schematic Describing Patient Inclusion for Study of HIV-1 Viremia in M98–863 Patients (21 KB PDF)

Figure S2 Correlation between Pretherapy Plasma HIV-1 RNA Levels as Determined by Amplicor HIV-1 Monitor Assay and SCA Pretherapy samples from 157 participants were analyzed by both assays. 12 samples (triangles) had SCA values significantly lower than Amplicor and were excluded from further analysis. Remaining samples (*n* = 145, diamonds) were highly correlated (*r^2^* = 0.61, broken lines represent ± 0.5 log_10_ copies/mL from Y = X line); corresponding participants were included in subsequent analyses.
(32 KB PDF)

## Abbreviations

LPV/r: lopinavir/ritonavir
NFV: nelfinavir
NNRTI: non-nucleoside reverse transcriptase inhibitor
SCA: single copy assay
