# Supplementary material for: ART Suppresses Plasma HIV-1 RNA to a Stable Set Point Predicted by Pretherapy Viremia
Source: PLoS Pathog. 2007 Apr 6;3(4):e46. doi: 10.1371/journal.ppat.0030046 (PMC1847689; doi:10.1371/journal.ppat.0030046)

Supplemental Figure 1. Subjects from trial M98-863 studied by longitudinal analysis

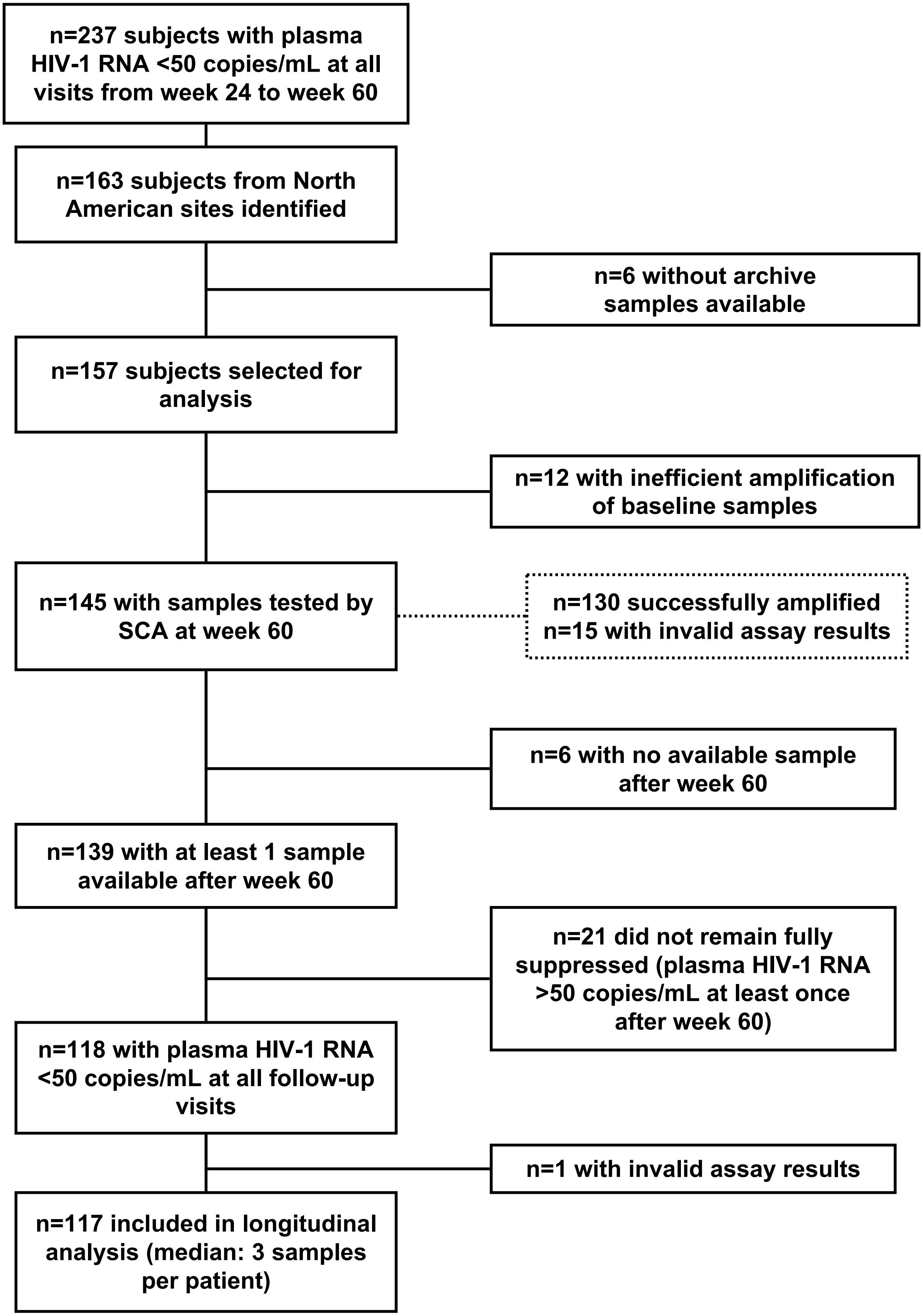

Supplement: Supplementary file 1 [file ppat.0030046.sg001.pdf]
