# Supplementary material for: ART Suppresses Plasma HIV-1 RNA to a Stable Set Point Predicted by Pretherapy Viremia
Source: PLoS Pathog. 2007 Apr 6;3(4):e46. doi: 10.1371/journal.ppat.0030046 (PMC1847689; doi:10.1371/journal.ppat.0030046)
Supplement: Supplementary file 2 [file ppat.0030046.sg002.pdf]

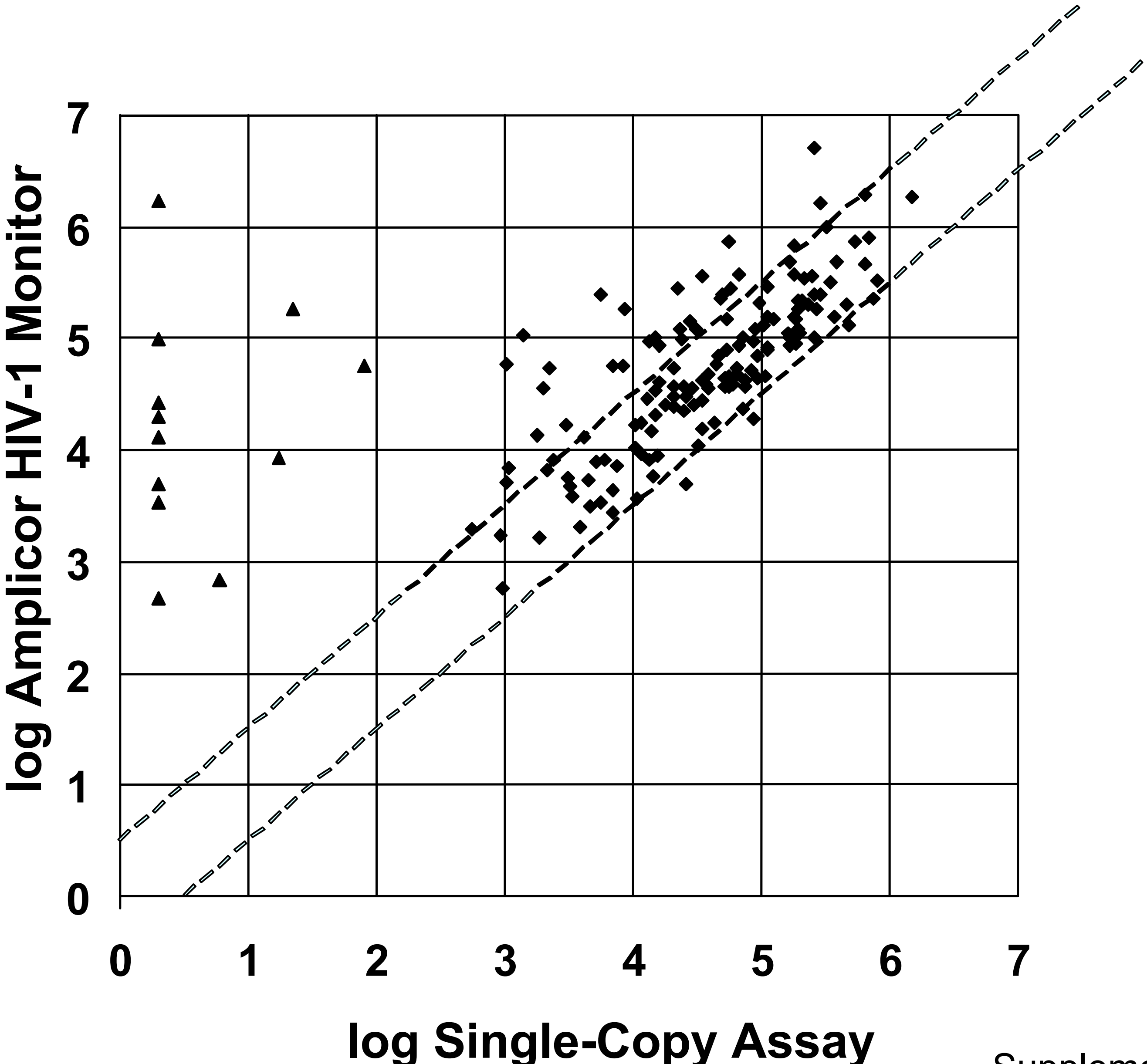

Supplemental Figure 2
